# Human gut strains of *Desulfovibrio piger* exhibit spontaneous induction of multiple prophages

**DOI:** 10.1128/aem.01917-25

**Published:** 2025-11-26

**Authors:** Melinda J. Mayer, Lizbeth Sayavedra, Kathryn Gotts, Nichola Wong, Henry Whiley, Marnie Barham, Arjan Narbad

**Affiliations:** 1 Quadram Institute Bioscience 7308, Norwich, United Kingdom; Universidad de los Andes, Bogotá, Colombia

**Keywords:** sulfate-reducing bacteria, *Desulfovibrio piger*, bacteriophage, spontaneous prophage induction, horizontal gene transfer

## Abstract

Sulfate-reducing bacterium *Desulfovibrio piger* is a common member of the human gastrointestinal microbiome, associated with inflammatory conditions but also prevalent in healthy individuals. This suggests that lifestyle factors may shape its ecological role. We investigated prophage carriage and release in three new *D. piger* strains from healthy donors and strain FI11049 from a patient with ulcerative colitis. Sequencing revealed a larger genome in strain FI11455 (3.096 Mb) compared to FI11311 (2.985 Mb) and FI11458 (2.838 Mb), including a 154 kb megaplasmid which contained an 87 kb section with high similarity to the chromosome of strain FI11311, suggesting horizontal gene transfer between chromosomes and plasmids. This section encoded genes involved in DNA replication, transcription, and recombination, as well as protein folding and modification, defense, and phage proteins. Strain FI11049 showed less than 95% similarity to other *D. piger* strains but shared similar prophages with them. Each strain carried four to five predicted prophages, ranging from 30 to 60 kb, which clustered into four groups, with at least three groups per strain. Although the prophages had no nucleotide similarity to known phages, genes for lysis, integration, regulation, and structural proteins were identified, and three groups contained Mu-like proteins. Electron microscopy and PCR of mitomycin C-induced supernatants confirmed the release of tailed bacteriophage particles and capsids of multiple prophages. Similar results were demonstrated from uninduced samples, indicating spontaneous prophage release. Host defense systems were widespread, and cross-infections failed to identify suitable hosts in related strains and species. This is the first evidence of prophage release in gut-associated *Desulfovibrio*, with implications for gene transfer in the gut.

**IMPORTANCE:**

Gastrointestinal health has a significant impact on quality of life, and increasing profiling of the gut microbiome is identifying key players involved in disease states. However, evidence of the association of sulfate-reducing bacteria with pathologies, such as inflammatory bowel disease and colorectal cancer, conflicts with their prevalence in healthy subjects. Investigating the ecology of *D. piger* in the gut may be key to answering if and why it can be harmful and could inform future interventions. Here, we show that gut-associated *D. piger* strains carry multiple prophages, some of which are spontaneously released as bacteriophage particles in culture. Our results pave the way for future work to understand prophage release in gut conditions and its effects on *D. piger* populations.

## INTRODUCTION

Sulfate-reducing bacteria (SRB) are a polyphyletic group with the ability to obtain energy via dissimilatory sulfate reduction, producing the toxic gas H_2_S (1). They exist in a wide range of environments, including the gastrointestinal tract (2, 3). *Desulfovibrio* is a prevalent SRB genus in healthy human fecal and colonic samples, with *Desulfovibrio piger* being the most commonly isolated species (4–6). *Desulfovibrio* has been associated with conditions including inflammatory bowel disease, disease severity in Parkinson’s disease, metabolic syndrome, and colorectal cancer (3, 7–9). Fecal H_2_S concentration and sulfate reduction rates were found to be higher in ulcerative colitis (UC) patients than healthy donors, and several studies have demonstrated a link between inflammatory gut conditions and *Desulfovibrio* abundance, including *D. piger* (3, 5, 10). However, other studies have observed the opposite effect (11), and a high incidence in healthy subjects indicates that *D. piger* carriage is not directly detrimental. A recent study utilized *D. piger* as part of a probiotic treatment, supporting butyrate production from *Faecalibacterium prausnitzii* by cross-feeding, with no noted adverse effects in humans (12). Interactions with other members of the gut microbiota might be involved with negative effects on the host, and diet may also play a role (6, 7, 13). For example, co-culture with *D. piger* decreased the susceptibility of *Clostridium difficile* to metronidazole, and the authors’ hypothesis that *D. piger* created a metal-limited environment via H_2_S production could have implications for many other gut microbes (14). Thus, the lifestyle of *D. piger* in the gut and its interactions with other strains and species may have important effects on human health.

Bacteriophages are viruses of bacteria and are characterized by huge diversity and prevalence (15–17). Temperate bacteriophages can exist in bacteria as prophages, either as episomal genetic elements or integrated into the bacterial chromosome as lysogens (18, 19). A range of signals or environmental conditions can induce the lytic cycle, producing infectious virions released by host lysis (20). A high proportion of gut bacteria are lysogens, and temperate phages can represent a major part of the gut virome (21–24). Poly-lysogeny, cryptic phages, and prophage remnants are also common features of bacterial genomes (22, 23, 25–27). Prophages can comprise a significant percentage of the genome, but this burden is countered by benefits, including protection from phages, competitive advantage against sensitive strains, opportunities for horizontal gene transfer (HGT), improved biofilm formation, resistance to stress, and carriage of auxiliary metabolic genes or virulence factors (19, 21, 27, 28). The impacts of temperate phages on bacterial hosts, prey, the wider bacterial community, and the human immune system are thought to make a major contribution to the ecology of the gut microbiome (29). The virome has also been shown to change or be distinct in disease states, with changes not necessarily being reflected in bacterial host populations (17, 30, 31).

Tailed bacteriophages or phage-like particles of *Desulfovibrionaceae* have been discovered through infection of environmental strains (32–34) or by induction with mitomycin C or UV light (35–37). Genome analysis enables further prophage discovery (38–41). A bioinformatic search of *Desulfovibrio* has found frequent poly-lysogeny with different forms of tailed phages, including in *D. piger* FI11049 (40).

The conflicting reports of the association of *D. piger* with health or disease suggest that particular conditions, such as population density, strain-specific characteristics, or interactions with the microbiome or host, could be involved in determining whether it acts as a harmless commensal or a pathobiont. Prophage carriage and release may be an important part of its lifestyle, with potential impacts on population sizes, HGT, and competition. Bacteriophages can also be potent tools for therapeutic intervention to target problematic strains. In this study, we examined prophages in three novel strains of *D. piger* isolated from healthy human guts and in UC strain FI11049 and demonstrated phage particle release during growth, providing direct evidence of prophage induction. These findings suggest that lysogeny is not a static state in *D. piger*, but a dynamic interaction between host and prophage, potentially influencing bacterial fitness, stress responses, and interspecies interactions in the gut environment. Understanding the role of prophages in *D. piger* could provide insights into how they affect its survival, metabolism, and adaptability, ultimately contributing to strategies for modulating its presence and activity in the gut microbiome.

## RESULTS

### New human gut *D. piger* isolates are distinct from *D. piger* FI11049 and contain a novel megaplasmid

Three novel strains of *D. piger* were isolated from healthy human fecal samples and sequenced using short- and long-read technology. Genomes of strains FI11311 and FI11458 assembled into single circular chromosomes of 2,985,077 bp and 2,837,551 bp, respectively, while strain FI11455 assembled into two circular contigs: a chromosome of 2,941,403 bp and a predicted megaplasmid of 154,448 bp (Table 1). The GC contents of the chromosomes ranged from 62.6 to 63.1%, but the GC content of the megaplasmid was notably lower at 56.7%.

**TABLE 1:** *D. piger* genome statistics

*D. piger* strain	No. of contigs	Genome size (bp)	GC content (%)	No. of CDS^*a*^	RNA^*a*^	Completeness/contamination/ strain heterogeneity (%)^*b*^	Accession no. (NCBI GenBank)
FI11311	1	2,985,077	62.8	2,614	75 tRNA, 19 rRNA, 10 ncRNA	99.29/0.59/0	CP189842
FI11455	2	3,095,851	1: 62.6	2,697	77 tRNA, 19 rRNA, 9 ncRNA	99.41/0.59/0	CP169274
			2: 56.7				CP169275
FI11458	1	2,837,551	63.1	2,442	71 tRNA, 21 rRNA, 5 ncRNA	98.70/0.59/0	CP169273

^*a*^ Annotation statistics from the NCBI Prokaryotic Genome Annotation Pipeline.

^*b*^ From CheckM.

**Fig 1:**
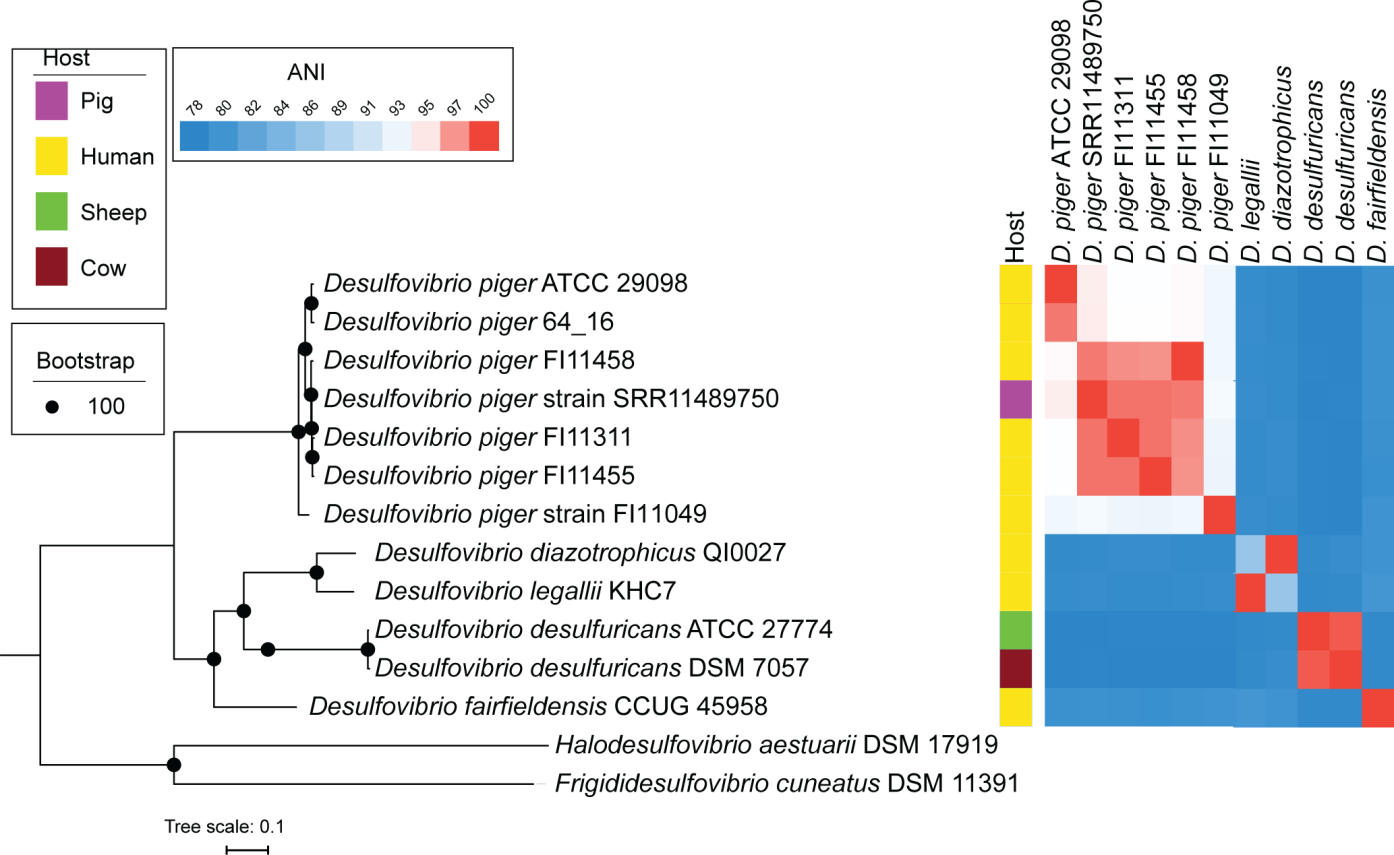
Phylogenomic reconstruction of *D. piger* and its closest relatives shows strain FI11049 may be a different species. The bootstrap support for all partitions was 100. Branch lengths are proportional to the number of substitutions per site. The scale bar represents the average substitutions per site.

**Fig 2:**
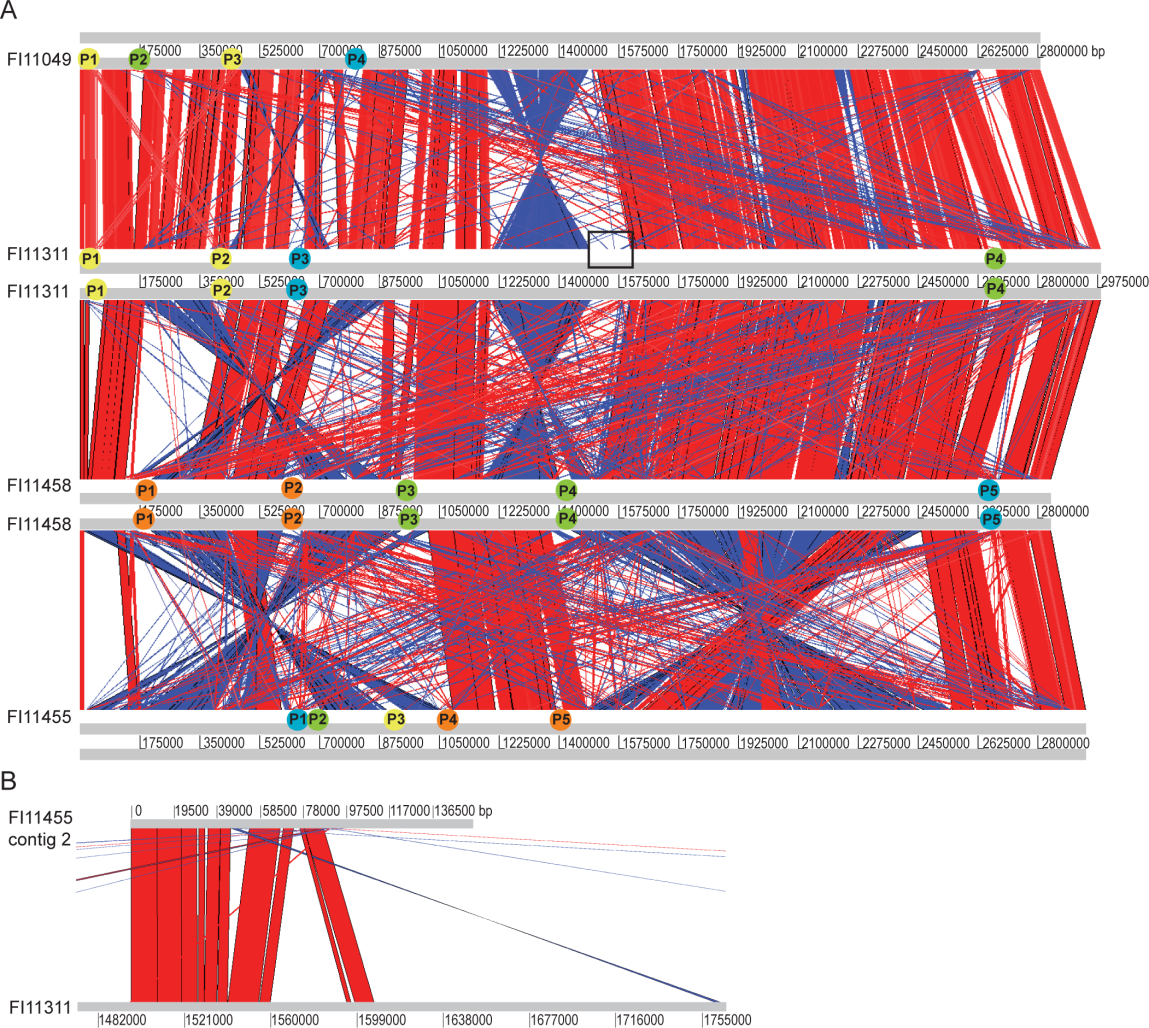
Nucleotide comparison of *D. piger* sequences identifies prophage locations and indicates plasmid-chromosomal exchange. (**A**) Comparison of chromosomes of strains FI11049, FI11311, FI11455, and FI11458 shows locations of predicted prophages, which are marked and colored by group—green, group A; yellow, group B; cyan, group C; and orange, group D. The section of FI11311 with similarity to the FI11455 megaplasmid is boxed. (**B**) Comparison of FI11455 megaplasmid with the boxed section of the FI11311 chromosome.

Genomes of these *D. piger* strains share high nucleotide homology with each other, with the human strain ATCC 29098, and with metagenome-assembled genomes (MAGs) from microbiomes of the human gut (*D. piger* 64_16) and pig colon (*D. piger* SRR11489750) (Fig. 1 and 2A). However, the human UC isolate FI11049, previously identified as *D. piger* (42), had only 93% average nucleotide identity (ANI) with these strains. This falls below the established 95–96% ANI threshold for species definition (43), suggesting that FI11049 represents a distinct species.

Annotation of the strain FI11455 megaplasmid did not identify *ori* genes, but a predicted RepB family plasmid replication initiator protein and a ParA family plasmid partitioning protein were reported (Data Set S1; Table S1). Blastn analysis and genome alignments showed little sequence similarity to the FI11455 chromosome or to strains FI11458 or FI11049, but half of the plasmid exhibited high nucleotide conservation with the FI11311 chromosome (Fig. 2B). This section (from 1,496,272 to 1,606,933 bp) appeared to be absent in strains FI11458 and FI11049 (Fig. 2A) and has a lower GC content (55.7%). Genome annotation shows predicted transposases at each end of the conserved section (Data Set S1; Table S1). Both sections of the megaplasmid exhibited only short regions of homology with other *D. piger* and *Desulfovibrio* spp. genomes, or with MAGs of Caudoviricetes partial genomes, with no notable homology identified in other lower GC-content species. Most of the 160 coding sequences were annotated as hypothetical proteins. Among those with a predicted function, many were associated with DNA synthesis, replication, transcription, and recombination. Several phage proteins were also predicted, but these were single or in small, isolated groups. In addition to toxin/antitoxin genes and tRNAs, Bakta annotation identified some of the genes involved in cobalamin biosynthesis in the part of the megaplasmid which did not show strong nucleotide homology to strain FI11311, encompassing cobalt transporters CbiM and CbiQ and cobalamin biosynthesis protein CbiL, followed by a transport-associated gene that Prokka annotated as cobalt import ATP-binding protein CbiO. All three new strains contain many of the genes required for cobalamin synthesis, but *cbiMLQ* is not present on the chromosomes, although other cobalt transporters are annotated (Data Set S1). Further upstream are a flavin-dependent oxidoreductase/monoamine oxidase, a Rid family detoxifying hydrolase, and a major facilitator superfamily transporter. ThiS and ThiF family proteins were also identified, members of which are involved in sulfur transfer in thiamin biosynthesis, among other roles. Also of interest were genes for protein folding and modification, including the chaperone protein DnaJ, a GNAT family N-acetyltransferase, an OmpH-like outer membrane protein, and a predicted type III secretion system chaperone.

### *D. piger* gut strains carry multiple related prophages/prophage remnants

The new genomes and previously described strain FI11049 carried 4–5 predicted prophages and several prophage remnants (Data Set S1). Remnants and isolated phage genes were also identified on the FI11455 megaplasmid. Chromosome nucleotide comparison allowed estimation of prophage ends, either by their insertion into homologous sequences (14/18) or by similarity to a matching prophage (Fig. 2A). The majority (13/18) of prophages were inserted in or next to tRNAs (Table 2).

**TABLE 2:** Predicted prophage statistics

Prophage	Group	Genome size (bp)	GC content (%)	No. of ORFs^*b*^	RNA^*b*^	Insertion site^*a*^	Location (orientation)
FI11311_P1	B	44,671	61.9	64	2 ncRNA	Between HP and tRNA	27192-71862 (+)
FI11311_P2	B	49,967	59.2	75	1 ncRNA	Intergenic region after IS element into tRNA	384575-434541 (+)
FI11311_P3	C	46,306	59.5	62	3 tRNA	Between toxin/antitoxin and transfer-mRNA SsrA	609798-656103 (-)
FI11311_P4	A	39,911	64.0	61		In small ribosomal subunit Rsm22 family protein	2655029-2694938 (+)
FI11455_P1	C	46,310	60.6	67	1 ncRNA	Into tRNA, followed by 11455_P2	619018-665327 (-)
FI11455_P2	A	39,264	64.9	54	1 ncRNA	Following 11455_P1, ending at tRNA	665328-704591 (+)
FI11455_P3	B	46,493	61.6	63	1 ncRNA	Into tRNA group	899285-945778 (-)
FI11455_P4	D	30,753	63.8	43	1 ncRNA	After tRNA, before AmmeMemoRadiSam system protein	1058903-1089655 (+)
FI11455_P5	D	22,957	58.8	28		After tRNA, before eutC	1391968-1414924 (+)
FI11458_P1	D	41,737	61.9	60		Into tRNA group	167537-209273 (-)
FI11458_P2	D	60,135	59.5	85	1 tRNA	Between tRNAs and methyltransferase	591233-651367 (-)
FI11458_P3	A	37,789	62.5	54		Between symporter and glycosyltransferase	937985-975773 (+)
FI11458_P4	A	36,974	65.0	53		Between permease and flavodoxin	1401400-1438373 (-)
FI11458_P5	C	47,955	59.4	69	1 ncRNA	Into tRNA group	2622269-2670223 (+)
FI11049_P1	B	43,575	59.9	63	3 ncRNA	Between HP and tRNAs	24311-67885 (+)
FI11049_P2	A	38,949	62.8	58	1 ncRNA	Between ornithine carbamoyltransferase and IS element	148226-187174 (+)
FI11049_P3	B	43,352	61.9	57		Between aldoketo reductase and tRNA	430014-473365 (+)
FI11049_P4	C	58,041	58.7	65	3 tRNA	Into tRNA gene	768981-827021 (+)

^*a*^ Insertion sites were predicted using Prokka.

^*b*^ Prophage ORFs (>40 amino acids) and tRNAs were predicted by Prokka, Bakta, and BV-BRC; ncRNAs were predicted by Bakta.

**Fig 3:**
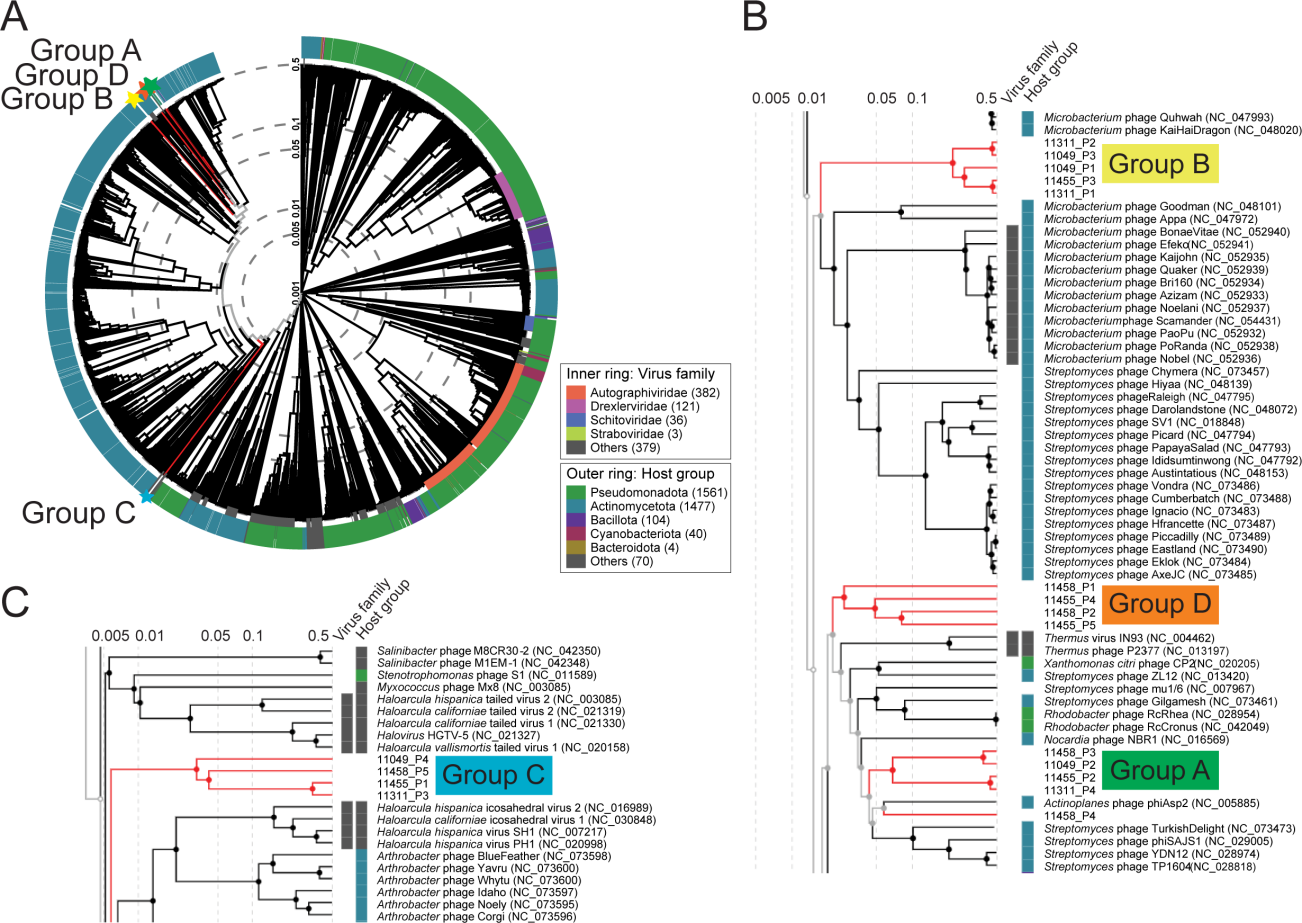
Location of predicted *D. piger* prophages on a viral proteomic tree shows prophages of groups A, B, and D cluster separately from group C. (**A**) Comparison to reference phage genomes (3359 sequences). (**B and C**) Detail of groups B, D, and A (**B**) and C (**C**) with surrounding phages.

Despite the lower ANI, predicted prophage regions from FI11049 shared intergenomic and proteomic similarity with those from FI11311, FI11455, and FI11458. The prophage sequences fell into four groups, A–D (Fig. 3; Fig. S1 and S2). Three groups (A, B, and D) were close neighbors on a proteomic tree, while group C was more distant (Fig. 3). Only strain FI11455 held all four prophage types (Fig. 2A). Intergenomic similarity between prophages of the same group was often higher between strains than within the same strain (Fig. S2). None of the prophages showed significant nucleotide similarity to other bacteriophages, but some homology to human-derived metagenome-assembled genomes (17) was observed (Table S2).

**Fig 4:**
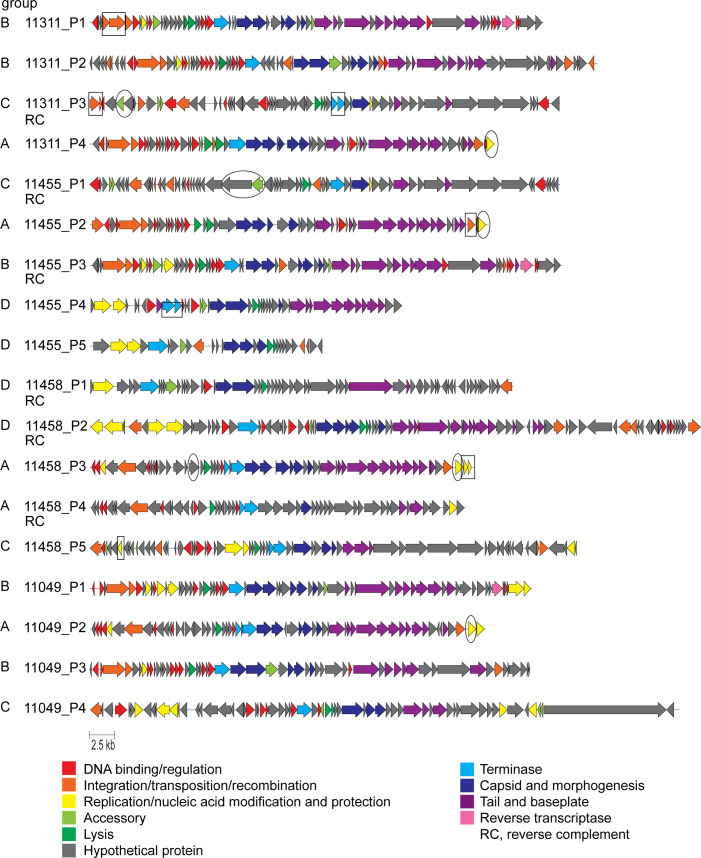
Annotation of *D. piger* prophages identifies structural and functional genes and indicates cryptic phages. Annotations on the left indicate prophage group (A–D). Predicted pseudogenes are boxed. Predicted defense systems are encircled.

Annotation identified capsid and tail structural and packaging proteins, lysis cassettes, transposases or integrases, transcriptional regulators/DNA binding proteins, and large terminase genes in most of the predicted prophages, but many proteins remain unidentified (Fig. 4; Table S3 to S20). The presence of sheath proteins and/or proteins with similarity to Mu or FluMu phages in members of groups A, B, and D suggested that these were myovirus-type phages, whereas group C had no Mu-like proteins and very few annotated structural tail proteins.

**Fig 5:**
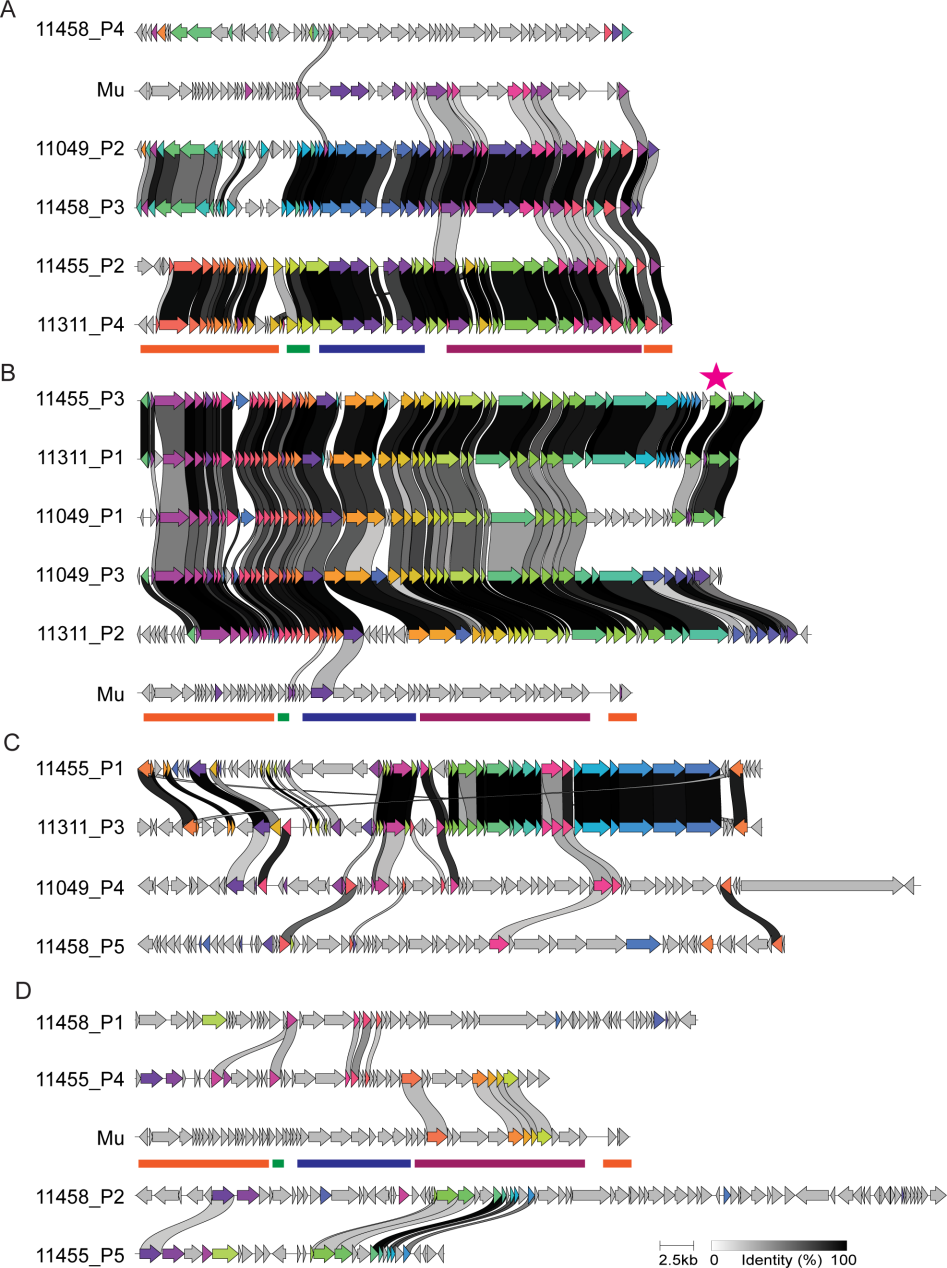
Translated prophage ORFs show homology within groups and with phage Mu. (**A**) Group A includes one outlier and two pairs of highly related phages with some conservation of structural, defense and regulatory proteins with Mu. (**B**) Group B shows high intergroup similarity with most variation in the tail region and low similarity to the Mu terminase. (**C**) Group C had no similarity to Mu and showed more variation. Few tail structural proteins were identified, and they showed some conservation. (**D**) Group D was also more disparate, and only 11455_P4 shared similarity with Mu tail proteins. ORFs are colored by homology group defined by clinker. Colored lines indicate functional regions of phage Mu (orange, integration, replication, modification, regulation; green, lysis; blue, capsid and morphogenesis; burgundy, tail). Star indicates retron-type reverse transcriptase.

Comparison of translated sequences showed that group B shared the highest similarity, while groups C and D were more diverse (Fig. 5; Data Set S2A through D). 11455_P5 shares homology with 11458_P2, but no tail proteins were identified, indicating a cryptic prophage (Fig. 4 and 5). Several other prophages had frameshifts or truncations in crucial genes (e.g., terminase, transposase), which may render them non-functional (Fig. 4; Tables S3, S5 to S7, S10, S14, S16). Comparison with phage Mu (44) confirmed limited amino acid identity with some predicted proteins in groups A (30–51%), B (30–42%), and D (31–57%), particularly the tail proteins (Fig. 5; Data Set S2A through D).

Predicted transposases and integrases were commonly located at prophage ends, but there were also transposases or mobile element proteins within some prophages (Fig. 4). A transposase from 11455_P3 had 36% identity with one in the megaplasmid, and three other megaplasmid proteins were related to proteins from the tail regions of group A, C, and D prophages (31–71% identity), indicating potential genetic exchange between prophages and plasmid (Data Set S2E).

No auxiliary metabolic genes were identified with confidence, and antibiotic-resistance and virulence factor-related genes were absent. However, most prophages carried accessory genes which may be linked to defense, including predicted toxin/antitoxin systems, single toxin domain or antitoxin genes, nucleic acid modification systems (e.g., restriction alleviation, methyltransferases, dCTP deaminase, Mom), and macro domain genes and ADP-ribosylglycohydrolases, which could have antitoxin activity (Tables S3 to S20).

Three prophages of group B contained retron-type reverse transcriptases, predicted to be part of diversity-generating retroelements (DGR), which can induce variation in a nearby target protein (45) (Fig. 4; Tables S3, S9 and S12). The reverse transcriptases, accessory proteins (containing domain cd16376 Avd_like), and target proteins (FGE-sulfatase domain-containing protein/phage tail fiber protein GpH) were highly similar between 11455_P3 and 11311_P1 (identities 99%, 100%, and 85% respectively; Fig. 5B; Data Set S2B), and the variable repeat covers a region of reduced amino acid homology between the target proteins (Fig. S3A). The 11049_P4 DGR has different predicted accessory and target proteins but with similar domains (Avd-like domain and FGE-sulfatase domain, respectively) (Fig. 5B; Fig. S3; Data Set S2B).

### Selected prophages release particles without induction

**Fig 6:**
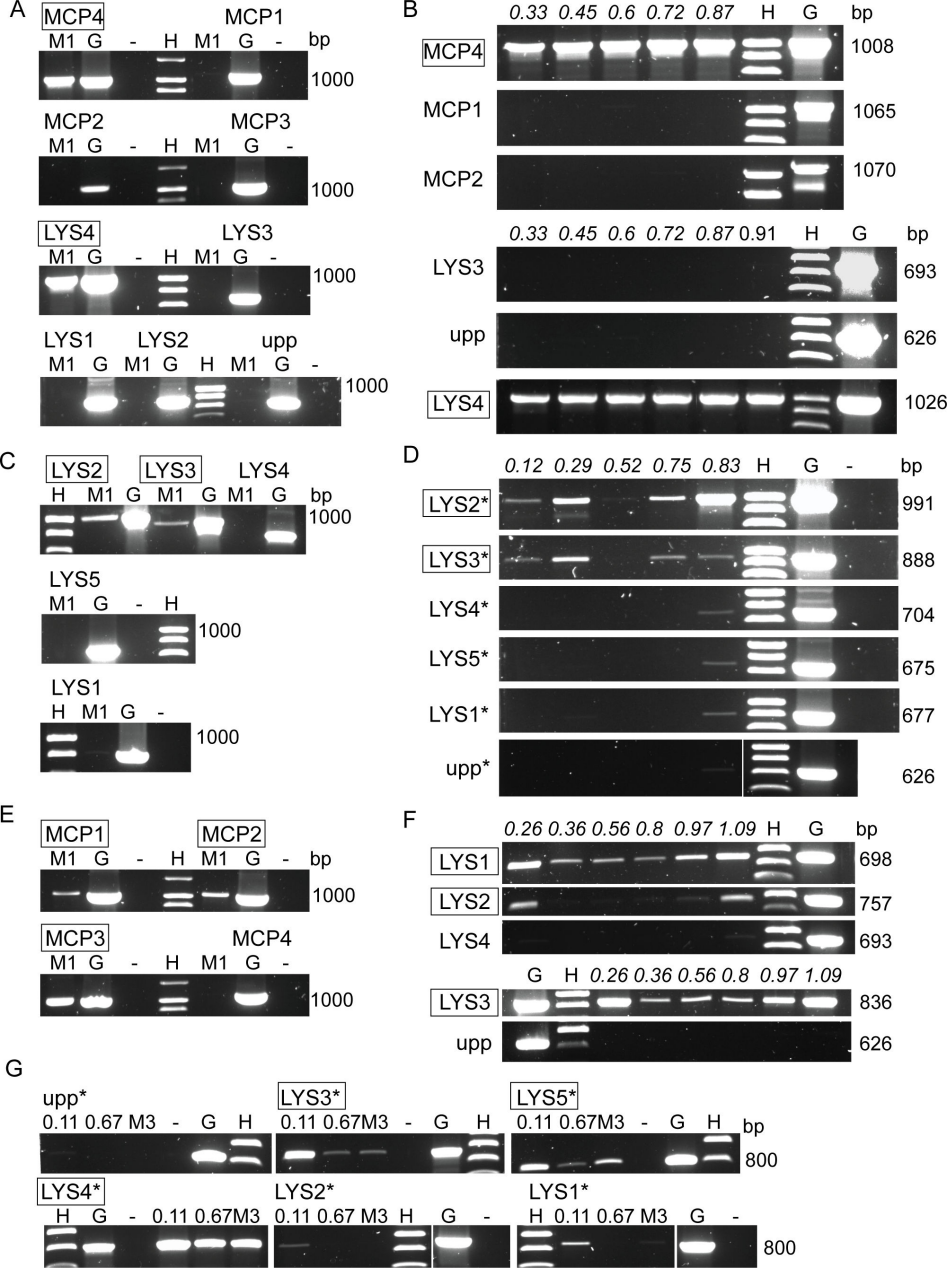
PCR amplification of prophage genes shows release with and without mitomycin C induction and at several growth stages. Primers amplified major capsid protein (MCP) or lysin (LYS) genes from *D. piger* strains FI11311 (**A and B**), FI11455 (**C and D**), FI11049 (**E and F**), and FI11458 (**G**). Primers targeting the bacterial gene *upp* were used to confirm the absence of bacterial DNA; only strong amplification from lysin or major capsid gene primers with no signal from *upp* was taken as an indication that prophage DNA was present. Primers indicating reliable prophage release are boxed. Primers to 11311_P4 (group A) showed particle release in induced supernatants (**A**) and throughout the growth period (**B**), but there was no evidence of release of prophages P1-P3. 11455_P2 (group A) was identified in induced samples (**C**) and at different growth stages (**D**). 11455_P3 (group B) was less prevalent, while 11455_P1, P4, and P5 were not detected reliably. Prophages 11049_P1, P2, and P3 (groups A and B) were released in all induced samples (**E**), but spontaneous release was less consistent (**F**, Fig. S5). Three prophages from FI11458 (groups A and C) were detected in most samples, with 11458_P4 being most prevalent (G). M1, M3, mitomycin C-induced supernatants; G, genomic DNA; −, PCR negative control; upp, bacterial primer control; H, DNA marker; numbers denote turbidities of uninduced supernatants at sampling, and italics indicate successive samples from the same culture. All reactions were run for 35 cycles except *, which were run for 30 cycles. Replicate experiments are presented in Fig. S5.

Prophage release was assessed using PCR analysis of PEG-precipitated supernatants, following DNase treatment to remove residual host DNA and heat treatment to disrupt capsids, alongside transmission electron microscopy (TEM). Mitomycin C induction did not affect the turbidity of cultures during growth or after overnight incubation compared to uninduced controls, suggesting that no large-scale lysis occurred (Fig. S4). PCR showed that encapsulated phage DNA was present in both induced supernatants and uninduced controls, indicating spontaneous prophage release (Fig. 6; Fig. S5). Two to three prophages were released for all strains except FI11311, where only P4 was identified, and phage DNA was seen at both early and late growth stages. Only 11311_P4 was consistently present in all samples and replicates tested, and no group D prophages were detected.

**Fig 7:**
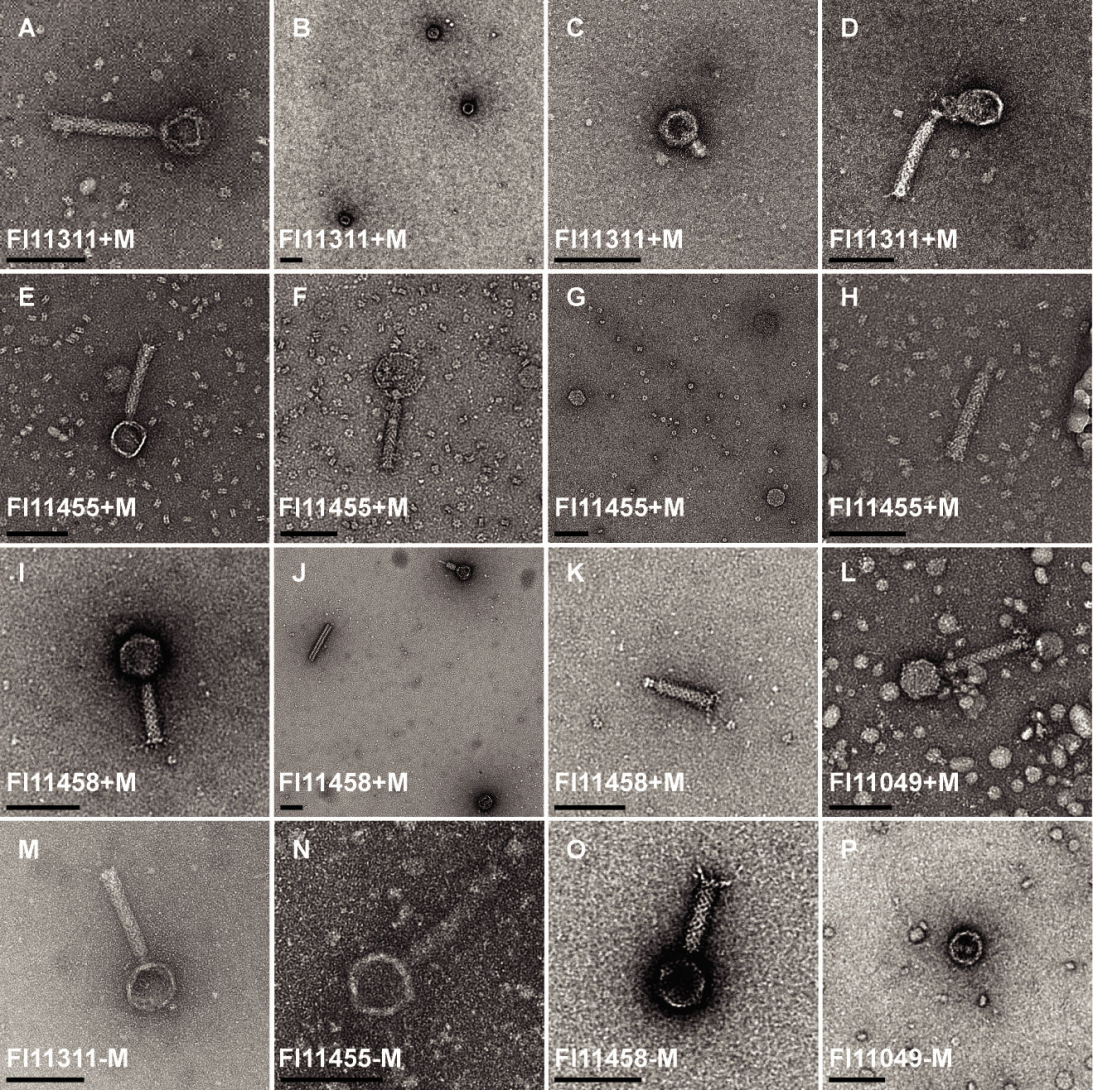
TEM analysis of *D. piger* phages. Particles were from PEG-precipitated supernatants induced with mitomycin C (+M, **A–L**) or uninduced (−M, **M–P**). Strain FI11311 exhibited long-tailed phages in induced samples (A; heads 51–62 nm, tails 113–124 nm by 15–23 nm, *n* = 6), free capsids (**B**), one short-tailed particle (**C**; head 43 nm, tail 21 nm by 15 nm), and evidence of particle damage and DNA release (**D**). FI11455 produced similar-sized tailed particles (E; heads 50–59 nm, tails 114–120 nm by 15–21 nm, *n* = 5), one particle with a larger but damaged head (F; c. 78 nm) and isolated capsids and tails (**G and H**). Phages from FI11458 had shorter tails (I; heads 56–62 nm, tails 78–86 nm by 16–20 nm, *n* = 14), with contracted particles and free heads, sheaths, and tails observed (**J and K**). Only one particle was found from two induced samples of FI11049 (L; head 60 nm, tail 126 nm by 19 nm). Similar particles were observed in uninduced cultures of FI11311 (**M**), FI11455 (**N**), and FI11458 (**O**), while only free heads were observed in uninduced FI11049 (**P**). Bar, 100 nm.

TEM identified whole bacteriophage particles from all four strains (Fig. 7), but they were rare, not observed in every induced sample, and appeared in similar numbers in untreated samples. One induction of FI11458 produced numerous particles. This sample did not exhibit a reduction in the final turbidity compared to the uninduced partner (Fig. S4E). Nearly all phage particles had a myovirus morphology with straight tails and tail fibers, and some contracted particles were seen. Bacteriophage dimensions from strains FI11311, FI11455, and the single FI11049 phage were similar, while FI11458 phages had shorter tails. One short-tailed particle from FI11311 could represent a podovirus or gene transfer agent (Fig. 7C), but no tail fibers were visible. Broken phages, isolated tails, sheaths, and capsids were also observed. Despite PCR indicating production of more than one phage group in strains FI11455, FI11458, and FI11049, only one particle size was visualized in each strain, although this may be due to the low number of phages seen.

### Cross-infection between strains and related species was not observed

We used unprecipitated mitomycin C-induced supernatants to investigate whether released prophages could infect other *D. piger* strains or related *Desulfovibrio* species. In addition to the four producer strains, six new *D. piger* strains isolated from human fecal samples, *D. piger* ATCC29098, and six closely related species—*Desulfovibrio simplex* DSM 4141, *D. legallii* DSM 19129, and human fecal isolates *D. fairfieldensis* FI11312, *Desulfovibrio intestinalis* FI11315, *D. desulfuricans* QI0028, and *D. diazotrophicus* QI0027—were tested to identify potential bacterial hosts. Cultures were either inoculated with induced supernatants or left untreated for 24 h (to assess whether infection reduced turbidity, indicating lysis). Both the filtered supernatants of these primary infections and the original mitomycin-induced supernatants were then tested by spot plaque assay. None of the infections caused a notable difference in growth patterns or final culture turbidity at 24 h (Fig. S6), and no plaques or areas of clearing were seen on lawns of potential host strains, indicating that either none of the tested *D. piger* strains or *Desulfovibrio* species were suitable hosts, or that the released phages were not fully functional or not numerous enough to cause large-scale lysis.

### Bacterial genomes carry multiple antiphage mechanisms and transposases

As infection attempts were unsuccessful, we searched for phage defense systems in the sequenced genomes. All four *D*. *piger* genomes encoded a number of potential mechanisms both for defense and anti-defense, based on Bakta annotation and analysis with DefenseFinder (Table S21). In addition to multiple toxin/antitoxin systems, DefenseFinder predicted 18 defense subtypes, three of which were prophage-located (RM_Type_II on 11455_P1, DarTG on 11311_P3, and Belisama on 11458_P3), and three anti-defense subtypes, one of which was located on four out of five group A prophages (Mom). CRISPR arrays were found only in strains FI11311 and FI11455, while FI11458 had three *cas* genes. Strain FI11455 had the most systems (16). All strains contained restriction-modification and SoFic systems, and at least one unique system, including Hma, PD-T5-5, HEC-01, DRT, DarTG, Paris, Belisama, and Menshen. Some systems were embedded in others, and some spanned regions containing unidentified genes, which may represent further unidentified embedded defense systems. Several systems are preceded or interrupted by transposases, especially of the IS5 family, notably the restriction-modification systems.

## DISCUSSION

Genome analysis of gastrointestinal *D. piger* strains identified multiple prophages, some of which were released as either full particles or capsids in both induced and uninduced samples. Mu-like long-tailed prophages dominated. All strains also included a representative of group C with few structural tail proteins, but podoviruses with tail fibers were not identified by TEM. We found no or low nucleotide similarity to known phages, a common characteristic of intestinal phages (23, 29). Prophages were often inserted in tRNA genes, whose high conservation makes them stable targets for integration for bacteriophages and mobile elements (27, 34, 41). As well as intact phage particles, we found tail-free capsids, tails, and damaged phages. Prophage-like gene transfer agents containing bacterial DNA have been described in *D. desulfuricans* (46), and phage-like tail particles (tailocins) have been visualized from *N. vulgaris* (36), but there was no genomic evidence of tailocins in *D. piger*.

Poly-lysogeny, where a single bacterial cell carries multiple prophages within its genome, is a common feature of gut microbes (22, 23, 25, 30). It is proposed to be widespread among bacteria (47) and can benefit bacterial fitness (48). Transposon-containing phage Mu can replicate and reintegrate into the chromosome (49), so similar prophages within the same strain may have evolved from duplication. However, although some strains contained two prophages of the same group, these either did not share notable intergenomic similarity or showed equivalent or higher similarity to prophages in other strains, suggesting transfer between *D. piger* strains. Previous work found that prophages from *Desulfovibrio* genomes were classifiable into subgroups, some represented in different species (40). Our study confirms that related prophages can be shared between strains with less than 95% ANI, indicating potential for HGT between *Desulfovibrio* species.

Several prophages carried mobile elements, which may contribute to HGT between prophages or with the bacterial genome, where mobile elements were common (50). Transposases and phage genes/remnants were also identified on the FI11455 megaplasmid. The section of this plasmid that showed high nucleotide similarity to the strain FI11311 chromosome was flanked by predicted transposase genes, supporting the hypothesis of HGT within and between *D. piger* strains. Isolated phage genes on the megaplasmid may also represent opportunities for phage evolution. This is particularly relevant for tail proteins, which could impact host range. Opportunities for host range modulation may also be provided by the diversity-generating retroelements identified in three prophages, some of whose target proteins showed similarity to tail fiber domains (51). The fact that the megaplasmid and some prophages show different GC ratios to the main chromosomes suggests that genetic material may also be obtained from other species. Megaplasmids have previously been described in *N. vulgaris* (41, 52), where poly-lysogeny and variation of prophage location between strains were also observed (41).

Prophage 11455_P5 is likely to be cryptic, while others have frameshifts or truncations in essential genes (e.g., terminase, transposase) which may render them inactive. Cryptic or defective phages can also have an impact on the host and be released spontaneously or induced (27). Many prophages carry genes with potential benefit to the host (15, 21, 29), including genes that affect the host phenotype and auxiliary metabolic genes (19), but we did not reliably detect these in *D. piger*. However, we found multiple potential defense systems on the *D. piger* prophages, with toxin/antitoxin and protection against restriction being the most common, as reported in other studies (25, 27, 28). Toxin/antitoxin pairs and single genes may also be involved in other functions such as toxin blocking or prophage activation (53, 54). Several ORFs predicted to relieve ADP-ribosylation were identified (55). Some potential bacterial defense genes, such as dCTP deaminase (56), were located on prophages with truncations or frameshifts - carriage of such beneficial genes could explain their retention as cryptic phages. Host defense systems were also widespread, which may have contributed to our inability to find a sensitive host among human gut isolates or related species, although inherently narrow host ranges or prophages conferring superinfection exclusion may also be factors. CRISPR-Cas systems were identified in several *Desulfovibrio* genomes, although not FI11049 (40). Many defense genes have been identified by co-location with others in “defense islands” (54, 57); the ORFs embedded in *D. piger* defense systems may represent novel candidates for future investigation.

Our results indicated that phage particles could be found throughout growth, and often more than one prophage was released. Spontaneous prophage induction from a subset of the population is a well-developed concept, and research has demonstrated several potential mechanisms as well as their impacts on bacterial populations (58). Several important intestinal genera show prophage release in the gut (22), and spontaneously produced phages will contribute to the gut virome and may have an effect on its stability and diversity (16, 24). Active phage production can confer a fitness benefit *in vivo* when competing with a phage-sensitive strain (26). Four prophages of intestinal *Enterocloster clostridioformis* were released spontaneously *in vitro* (25); lysogens of IBD-associated *Ruminococcus gnavus* also released viable phages (30), while spontaneous induction and co-induction of different prophages from the same cell were observed with Shiga toxin-encoding prophages (59). Excision/release of phages without induction was seen previously in environmental sulfate reducers *N. vulgaris* (34, 41) and *D. desulfuricans* (38).

In most instances, mitomycin C did not increase *D. piger* phage production or cause large-scale lysis. Phage-like particle induction from *N. vulgaris* also failed to decrease turbidity (36). It is possible that mitomycin C is not an appropriate induction agent for these prophages. In addition to induction via the host SOS response, further stimuli are being uncovered which may have more relevance *in vivo*, including inflammation, diet, antibiotics, oxidative stress, bacterial metabolites, and quorum sensing (20, 47). Multiple prophages may benefit from responding to different stimuli to avoid competition for host resources (47). Given that we identified encapsulated phage genes at several growth stages, it is unlikely that the trigger for *D. piger* prophage release is nutrient limitation or high population density. However, *in vitro* growth itself may be a stressful situation for this bacterium, which we found frequently challenging to culture and irregular in its growth patterns.

Yields of phage particles from both induced and uninduced *D. piger* strains were low. Longitudinal analysis of bacterial and viral metagenomic samples from a healthy human gut demonstrated low-level, continuous phage production, accompanied by high viral diversity (24). Such low spontaneous induction rates may be advantageous for lysogens, offering competitive benefits without compromising host viability (60). In this context, sustained but limited phage release could serve as a subtle defense mechanism against competing strains. However, the high level of infection resistance we observed among related *Desulfovibrio* strains and species raises questions about whether spontaneously released *D. piger* phages are capable of exerting meaningful ecological pressure. Resistance may be mediated by superinfection immunity from similar phages or by the array of defense factors identified. A further consideration is that the phage particles detected in this work may be defective and not capable of infecting other strains or have a low efficiency of infection. In any case, it is also possible that spontaneous induction may fulfill alternative roles, such as promoting biofilm formation via extracellular DNA release (61) or affecting virulence or HGT (58).

It has been proposed that the interactions between phages, bacteria, and the host have major impacts on the ecology of the gut microbiome (29). Regular lysis of a subpopulation may facilitate strain evolution by releasing genetic material, including chromosomes, phages, and megaplasmids. These findings highlight an interaction between *D. piger* prophages, lysogeny, and spontaneous phage induction, echoing observations in environmental sulfate-reducing bacteria. The extent to which prophages function as latent genetic reservoirs, modulators of bacterial fitness, or drivers of evolutionary adaptation in *D. piger* populations remains an open question. Future research should aim to understand the selective pressures that shape prophage activation in *D. piger*, as well as the potential consequences of phage-mediated interactions in the gut environment with the rest of the microbiota.

## MATERIALS AND METHODS

### Strains and growth conditions

Chemicals were from Merck unless stated otherwise. *Desulfovibrio* species were routinely grown in anaerobic conditions (5% CO_2_, 10% H_2_ in N_2_ at 37°C, Don Whitley, UK) in Hungate tubes in Postgate C medium (62), or Postgate C supplemented with 10 mL per L Thauer’s vitamin solution (63) and MOYLS4 trace elements solution (64) (PVT), or in a medium modified from SRB641 (6) (SRB641mod—pyruvic acid and malic acid replaced by 0.6% sodium lactate, with vitamins and trace elements as in PVT). For plates, agar was added at 1.5%, and plates were reduced 1 to 2 days before use. Cultures were maintained on 0.7% agar slopes at 4°C in Hungate tubes. Glycerols were produced from 10 mL grown cultures, pelleted by centrifugation at 1,700 × *g* and 4°C for 12 min, then transferred to Protect Select Anaerobe Cryopreservation tubes (Technical Service Consultants, UK), and stored at −80°C.

*D. piger* strains were isolated on Postgate C agar from fresh fecal samples from different healthy human donors obtained from the QIB colon model study (approved by the Quadram Institute Bioscience Human Research Governance Committee [IFR01/2015] and London-Westminster Research Ethics Committee [15/LO/2169], registered at https://clinicaltrials.gov/study/NCT02653001) in 2015 (strains FI11455, FI11458) or 2017 (FI11311), or from individuals over 60 years old in 2023 (MOTION study, IRAS 241617, approved by the QIB Human Research Governance Committee [QIB04-2018], registered as NCT04199195; strains QI0440, QI0441, QI0469, QI0470, QI0471, and QI0472). Research was performed in accordance with the Declaration of Helsinki and the participants provided their written informed consent. Species for host range studies were obtained from the DSMZ, Braunschweig (*D. piger* ATCC29098, *Desulfovibrio simplex* DSM 4141, and *Desulfovibrio legallii* DSM 19129) or from institute collections of human fecal isolates (*Desulfovibrio fairfieldensis* FI11312 and *Desulfovibrio intestinalis* FI11315 [H. Whiley, unpublished], *Desulfovibrio desulfuricans* QI0028, and *Desulfovibrio diazotrophicus* QI0027 [65]).

### Genome sequencing and analysis

Genomic DNA was isolated using the GenElute Bacterial Genomic DNA kit (Sigma-Aldrich) or cetyltrimethylammonium bromide-based extraction (66). Whole-genome sequencing was performed with short and long reads as described previously (65). Long reads were assembled with Flye (v.2.9) (67), and hybrid assemblies were created with Unicycler (v.0.5.0) (68). Both assemblies of FI11455 produced two contigs defined as complete. Flye assembly of FI11458 and FI11311 produced single fragments, and the final hybrid assemblies were confirmed and closed by read mapping with BWA (v.0.7.17) (69) and manual sequencing (Eurofins, Germany). Genome statistics and quality checks were performed with Quast (v.5.0.2) (70) and CheckM (v.1.2.0) (71) using the Deltaproteobacteria marker lineage (UID3218) on the Galaxy platform (72).

Genomes were annotated with Prokka (v1.13) (73) and Bakta (v.1.10.3 Database v.5.1) (74); predicted prophages were annotated using a 40 amino acid cut-off with Prokka, Bakta, and the Bacterial and Viral Bioinformatics Resource Center (BV-BRC) using the bacteriophage protocol (75), with incongruent start sites checked using Artemis (v.18.2.0) (76) and Blastp (77), and functions checked with Blastp or InterProscan (78). For phylogenomic tree construction, *D. piger* genomes and related species were retrieved from the BV-BRC, and a set of 500 single-copy core marker genes was identified and aligned using MAFFT (v.7.490) with codon and amino acid partitions (79). A maximum likelihood phylogenomic tree was reconstructed using RAxML (v.8.2.12) under the GTRCAT model, with 100 rapid bootstrap replicates (80). The final tree was visualized and annotated using iTOL (v.6.9.1) (81). Artemis comparison tool (ACT, v.18.2.0) (76) comparisons were run using Blastn with an algorithm window size of 640 (red, good match; white, low/no match; blue, inverted match). Prophage genome backbone alignments were performed in BV-BRC using Mauve (82) with FI11311_P4 as the reference. Prophage nucleotide sequences were compared using VIRIDIC (83), and proteomic trees were generated using ViPTree (v.4.0) (84). Proteome comparisons were performed on separate groups and to *Escherichia* phage Mu (AF083977 (44) using clinker (CAGECAT_v.1.0) (85) with a minimum alignment sequence identity of 0.3. Predicted prophage sequences were compared to database sequences using NCBI Virus Blast (https://www.ncbi.nlm.nih.gov/labs/virus/vssi/#/) on 9 October 2024. Diversity-generating regions were analyzed with MyDGR (45). Amino acid alignments were performed with Clustal Omega in Geneious v.2022.1 (Biomatters Ltd.). Abricate (v.0.9.7, https://github.com/tseemann/abricate) was run with the CARD (86) and VFDB databases (87). Defense and anti-defense genes were assessed with DefenseFinder (57, 88) with the anti-defense option enabled.

### Prophage induction

Cultures (10 mL) were treated with 3 µg/mL mitomycin C (Merck) and incubated for 24 h before harvest. Turbidity (T) was measured in Hungate tubes with a turbidimeter CO8000 (Biochrom). Inductions were commonly performed with young cultures (T = 0.2–0.5), and the effect on growth was compared to uninoculated sibling cultures. Samples were centrifuged at 1,700 × *g* and 4°C for 12 min, and supernatants were filtered with 0.45 µm filters (Millipore). Samples for infection or plaque assay were stored at 4°C. Samples for TEM or prophage PCR were concentrated by polyethylene glycol (PEG) precipitation (89). After supernatant removal, pellets were consolidated by centrifugation for 1 min and then resuspended in SM buffer (89) overnight at 4°C (30 µL for TEM, 200 µL for PCR) before extraction with an equal volume of chloroform. For prophage release during growth, 2 mL samples were removed at intervals from the 10 mL culture, filtered, and PEG-precipitated as described above, with centrifugation at 16,000 × *g* for 20 min at 4°C. Mitomycin C inductions were performed at least three times.

### Prophage gene detection by PCR

Encapsulated genes were detected by PCR using an adaptation of existing methods (39, 90). Briefly, 17 µL PEG-precipitated supernatant was incubated with 1 µL RQ DNase and 2 µL RQ buffer (Promega) for 1 h at 37°C, followed by addition of 1 µL RQ stop solution. Capsids were broken at 95°C for 30 min, and 2 µL aliquots were used as templates with GoTaq G2 DNA polymerase (Promega), for either 30 cycles or 35 cycles to demonstrate gene absence, using genomic DNA (10 ng) as a positive control and multi-strain primers to the bacterial *upp* gene (uracil phosphoribosyltransferase, DESPIGER_0848) to confirm the absence of genomic DNA. Primers were obtained from Merck (Table S22) and dNTPs from Bioline. Samples that gave clear amplification of the *upp* gene, indicating bacterial DNA contamination, were not used further.

### Infections and plaque assays

Mitomycin C-induced supernatants (100 µL) were added to 10 mL overnight cultures, and infections were incubated for 24 h, with turbidity measured regularly in comparison to uninfected siblings. For cultures with high levels of iron precipitation (QI0440, QI0441, QI0469, and QI0472), bacterial densities were measured visually. Supernatants were harvested after filtration (0.45 µm) and centrifugation at 1,700 × g for 15 min at 4°C. For other *Desulfovibrio* species, 10 mL cultures at T 0.15–0.46 were split into 2 mL aliquots, inoculated with 20 µL mitomycin C-induced supernatants or left untreated, and incubated for 24 h before a final turbidity measurement. Plaque assays were performed using a modification of a previous method (33) using PVT soft agar overlays with 10 µL spots of mitomycin C-induced supernatants or filtered infections, incubated for 24 to 48 h.

### Transmission electron microscopy

PEG-precipitated supernatants from mitomycin C-induced and uninduced supernatants were viewed by TEM after negative staining with uranyl acetate using TEM grids (400 mesh, carbon-coated, copper grids, Agar Scientific) prepared by glow-discharging with a Leica ACE200 (Leica Microsystems). Suspensions (10 µL undiluted or diluted 1:1 with dH_2_O) were left for 1 min on grids, excess liquid wicked off using filter paper, and 10 µL 2% aqueous uranyl acetate (BDH Chemicals) overlaid for 1 min before further wicking and air drying. Grids were imaged (FEI Talos F200C TEM (Thermo Scientific)) at 200 kV with a Gatan OneView camera (Gatan) for 20–30 min each. Bacteriophage measurements were made with ImageJ (v.1.54, Fiji).

## Data Availability

Nucleotide sequences of strains FI11311, FI11455 (chromosome and plasmid), and FI11458 have been submitted to NCBI under accession numbers CP189842, CP169274-CP169275, and CP169273, respectively. Bakta annotation files are available as supplemental material.

## SUPPLEMENTAL MATERIAL

Supplemental material aem.01917-25-s0001.pdf Figures S1 to S6; Zenodo link to Data Set S1.

Supplemental tables aem.01917-25-s0002.xlsx Tables S1 to S22.

Data Set S2 aem.01917-25-s0003.xls Translated protein identities from Clinker analyses.

ASM does not own the copyrights to Supplemental Material that may be linked to, or accessed through, an article. The authors have granted ASM a non-exclusive, world-wide license to publish the Supplemental Material files. Please contact the corresponding author directly for reuse.
