# Supplementary material for: Human gut strains of Desulfovibrio piger exhibit spontaneous induction of multiple prophages
Source: Appl Environ Microbiol. 2025 Nov 26;91(12):e01917-25. doi: 10.1128/aem.01917-25 (PMC12724379; doi:10.1128/aem.01917-25)
Supplement: Supplemental material — Figures S1 to S6; Zenodo link to Data Set S1. [file aem.01917-25-s0001.pdf]

Supplementary Figures and Data

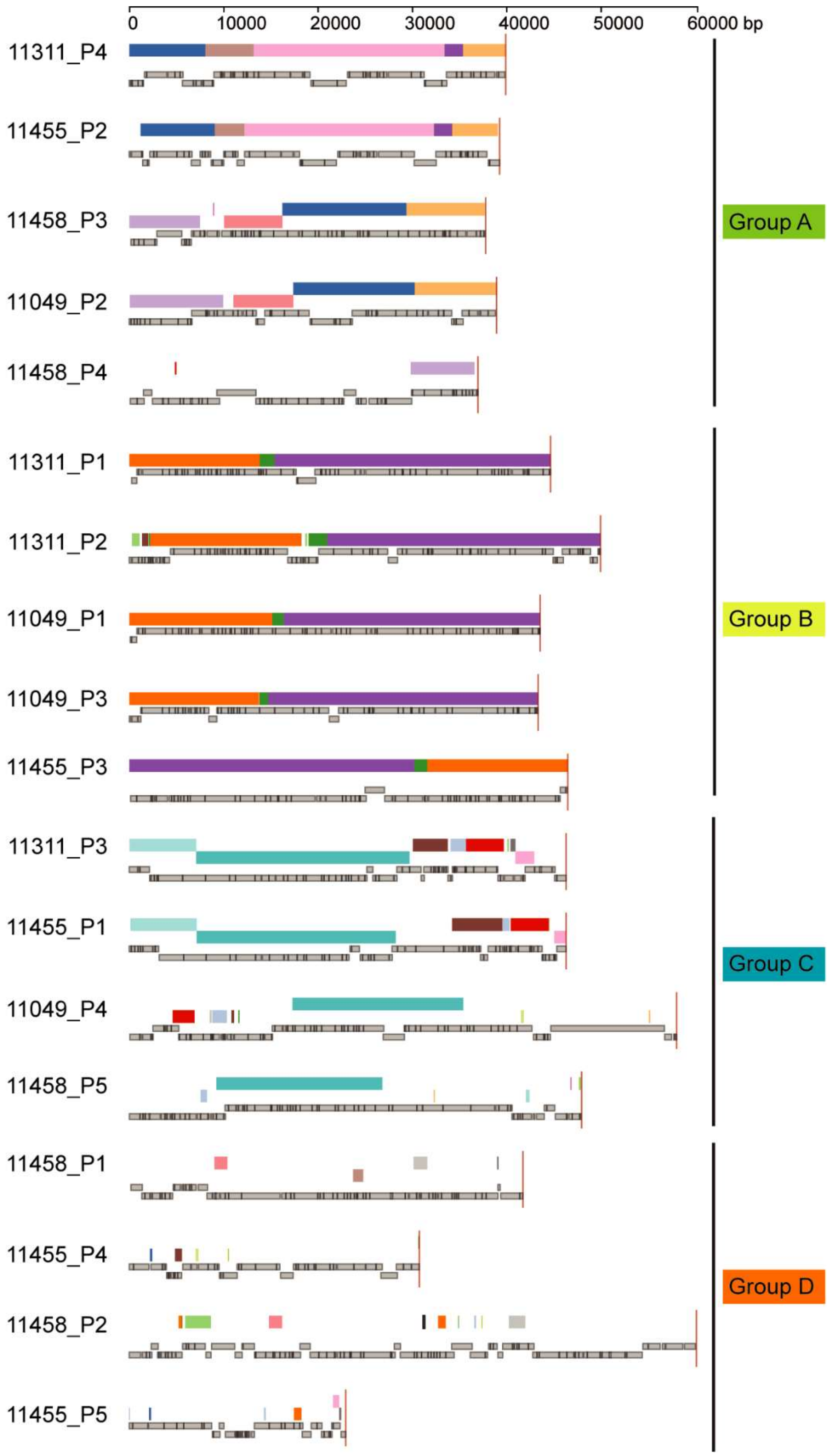

Figure S1 Simplified Mauve backbone analysis of *D. piger* predicted prophages.

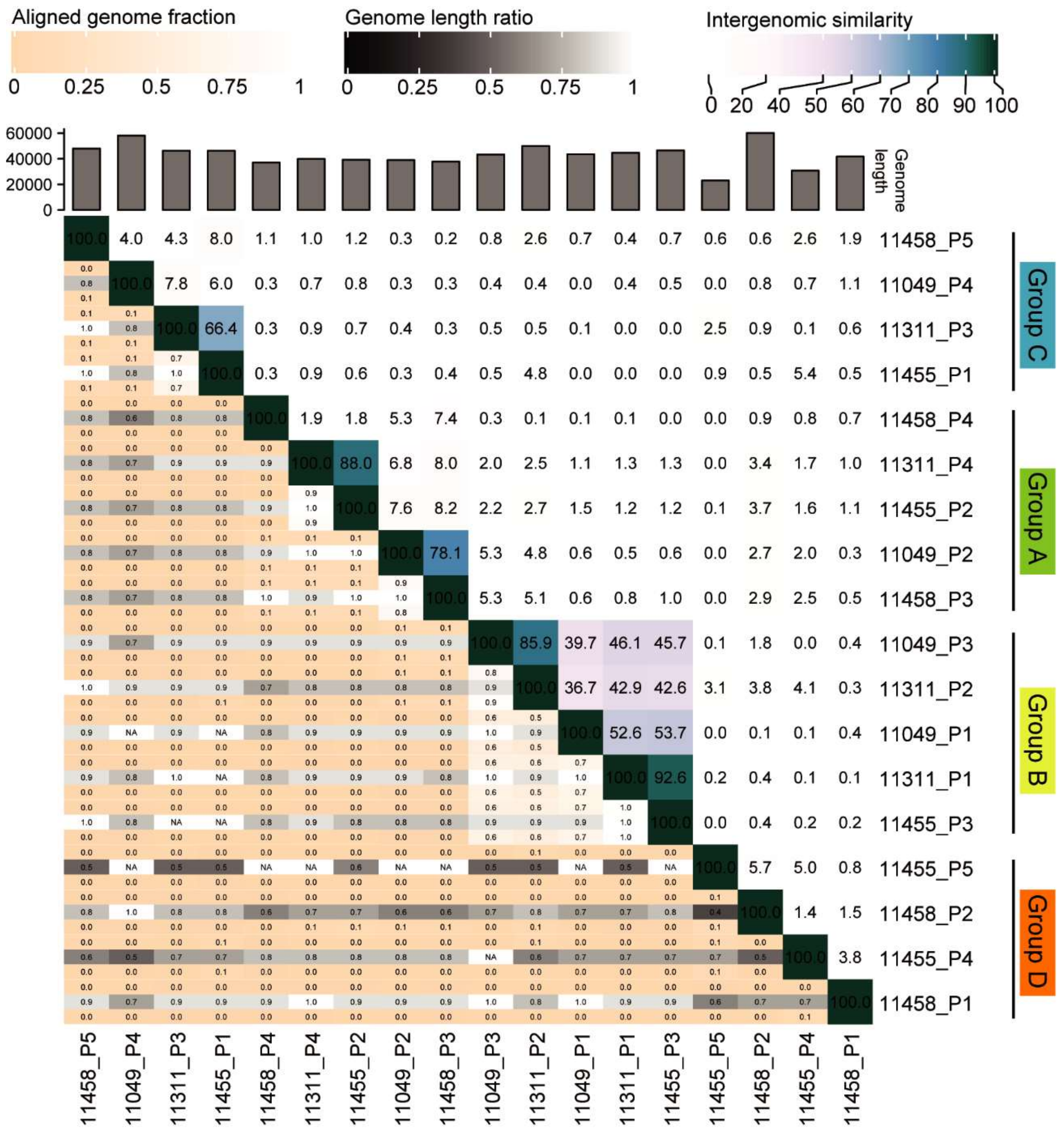

Figure S2 Intergenomic similarities between predicted *D. piger* prophages calculated using VIRIDIC. Figures to the right represent similarity values of each genome pair, figures to the left show (top to bottom) the fraction of genome 1 aligned, the genome length ratio, and the fraction of genome 2 aligned.

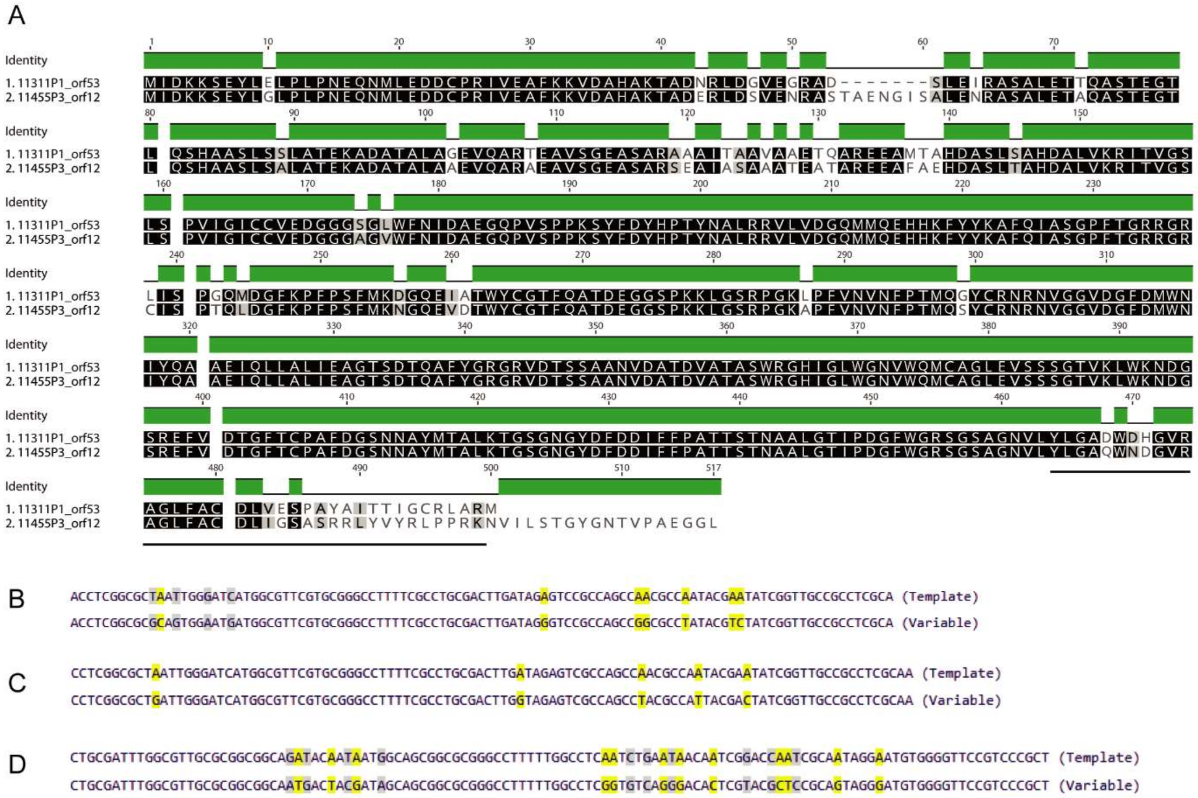

Figure S3 Predicted prophage diversity generating elements identified by MyDGR. A, alignment of predicted target proteins from 11311_P1 and 11455_P3, area of 11455_P3 variable region is underlined; **B**, template ((-)4614-4719) and variable ((-)6532-6637) regions of 11455_P3; **C**, template (40219-40323) and variable (38302-38406) regions of 11311_P1; **D**, template (38050-38168) and variable (37406-37524) regions of 11049_P1. A-to-N substitutions are in yellow, other substitutions in grey.

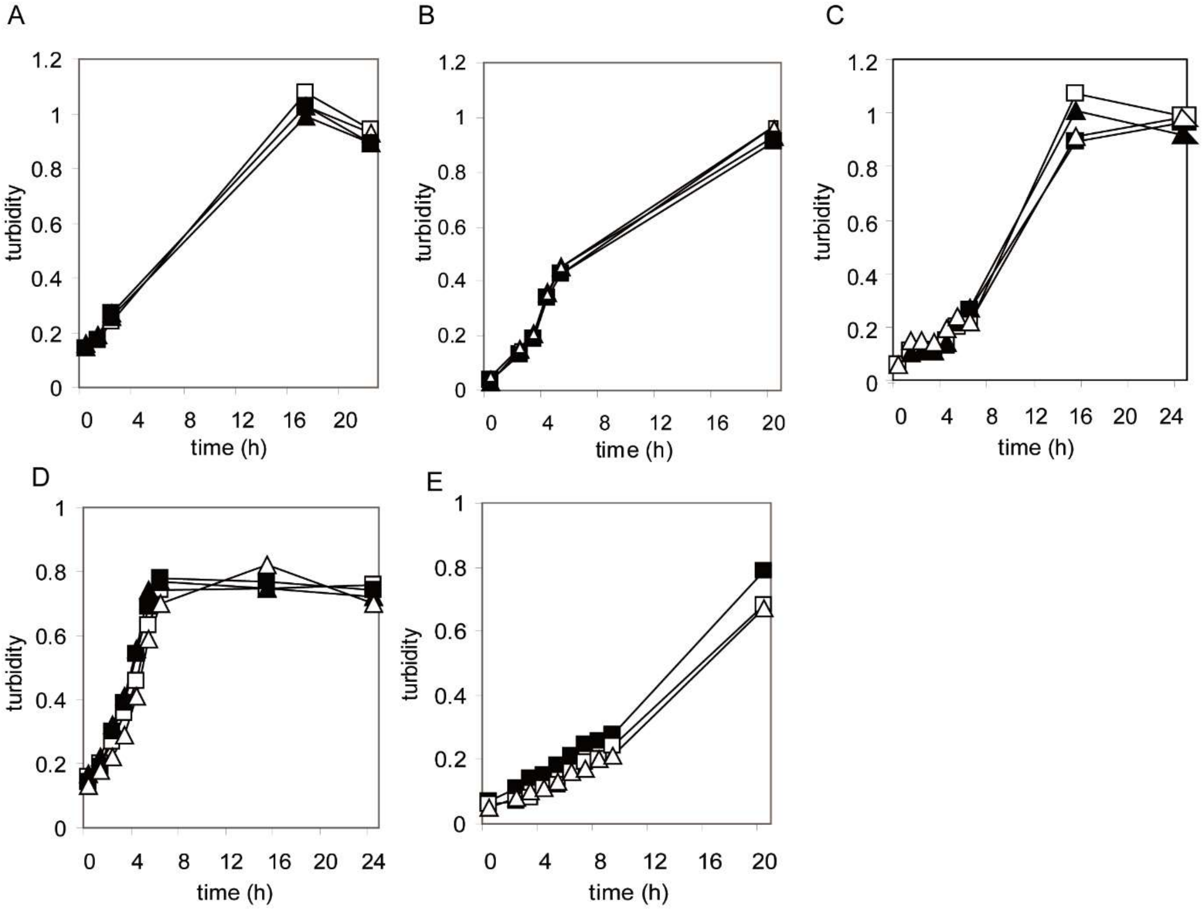

Figure S4 Growth during mitomycin C induction of *D. piger* strains. **A-D**, four cultures of each strain were either induced at turbidity 0.1-0.25 (filled symbols) or left uninduced (empty symbols) then incubated for 20-24 h. **A**, FI11311; **B**, FI11455; **C**, FI11458; **D**, FI11049; **E**, single induced culture of FI11458 which produced a high yield of phage particles compared to two uninduced sibling cultures. Triangles or squares represent sibling cultures with similar turbidities at induction.

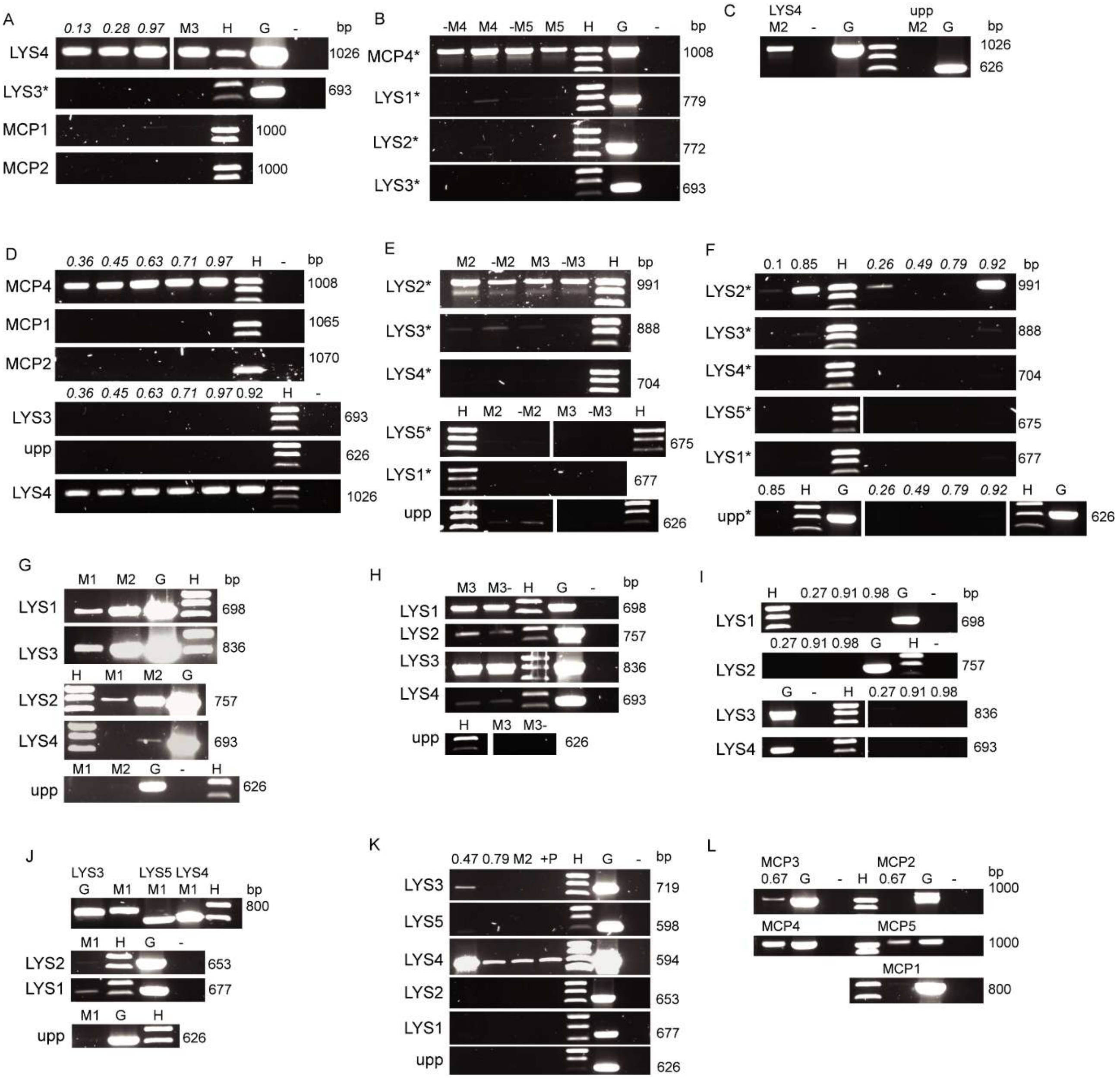

Figure S5 PCR amplification of prophage genes from mitomycin C-induced and uninduced supernatants. Primers amplified major capsid protein (MCP) or lysin (LYS) genes from *D. piger* strains FI11311 (**A-D**), FI11455 (**E, F**), FI11049 (**G-I**) and FI11458 (**J-L**). M, mitomycin C-induced supernatant; -M, uninduced sibling culture; G, genomic DNA; -, PCR negative control; upp, bacterial primer control; H, DNA marker; numbers denote turbidities of uninduced supernatants at sampling and italics indicate successive samples from the same culture. All reactions were run for 35 cycles except *, 30 cycles.

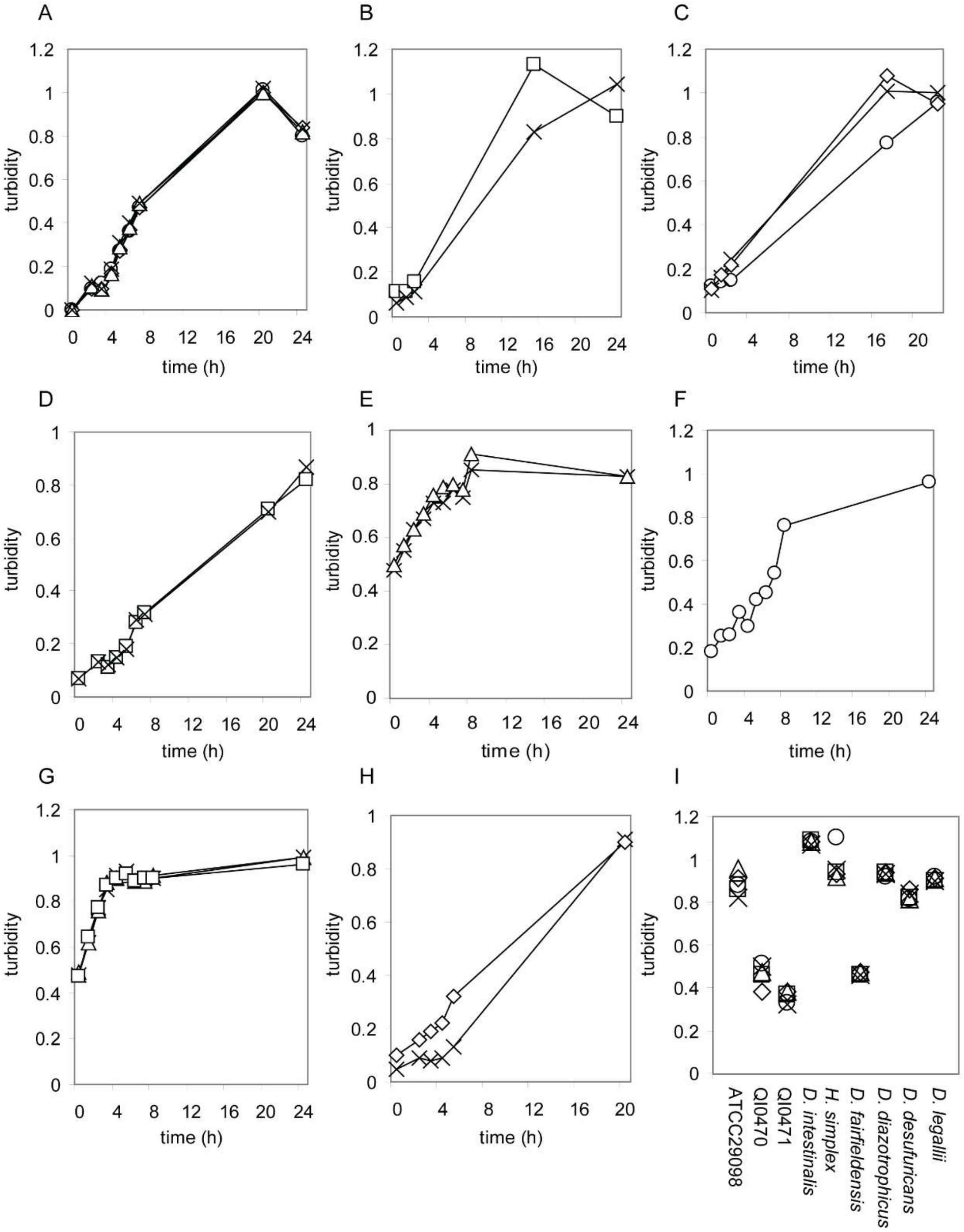

Figure S6 Infection of *D. piger* strains with mitomycin C-induced supernatants. **A-H**, cultures of each strain were infected with mitomycin C-induced supernatants from strain FI11311 (□), FI11455 (Δ), FI11458 (◊), FI11049 (○) or left untreated (x) for 20-24 h. **A**, FI11311; **B, C**, FI11455; **D-F**, 11458; **G, H**, FI1049; **I**, turbidities at 24 h from infections of *D. piger* strains ATCC29098, QI0470, QI0471 and related species compared to uninfected cultures. Turbidities of cultures of *D piger* QI0440, QI0441 QI0469 and QI0472 were saturated due to iron precipitates, but cultures showed no visible differences in bacterial density with or without infection.

Supplementary Data 1 Bakta annotation of *D. piger* FI11455; *D. piger* FI11311; *D. piger* FI11458, https://zenodo.org/records/15424475?token=eyJhbGciOiJIUzUxMiJ9.eyJpZCI6IjBiYzY5OTI1LWMwZTktNGFhNy05MDZiLWQ5NmQwM2M1ZDBjMiIsImRhdGEiOnt9LCJyYW5kb20iOiIzNWEzNTc3OGZmODY0ZGM3OTJjMTUzYjJhZTJhY2MyNyJ9.wE8NqZOXjlCwjDZATcevtSCEslXWyR852RhXyVfdjli3HXr6IFBRZdcbHUiiArE1WHfN7cgpxnK96xNIiYM60w
